# Effects of Acorn Flour Addition on Baking Characteristics of Wheat Flour

**DOI:** 10.3390/foods14020190

**Published:** 2025-01-09

**Authors:** Emilia Szabłowska, Małgorzata Tańska

**Affiliations:** 1 Department of Food Technology and Safety, Faculty of Health Sciences, University of Lomza, 18-400 Łomża, Poland; eszablowska@al.edu.pl; 2 Department of Plant Raw Materials Chemistry and Processing, Faculty of Food Sciences, University of Warmia and Mazury in Olsztyn, 10-726 Olsztyn, Poland

**Keywords:** acorns, baking value, rheological properties, pasting properties, baking test

## Abstract

This study aimed to evaluate the impact of incorporating acorn flour (at levels ranging from 5% to 50%) on the baking properties of wheat flour (type 750). The assessment focused on key baking parameters, including fermentation properties, pasting behavior, and dough rheological characteristics such as farinographic and extensographic properties. A laboratory baking test was conducted to compare the technological properties of wheat and wheat–acorn breads, assessing dough and bread yields, oven and total losses, bread volume, and crumb hardness. Additionally, the nutritional value of selected bread variants was established. The results indicated that flour mixtures with acorn flour exhibited a significantly reduced capacity to retain gases produced during fermentation (by up to 92%) and increased resistance to gelatinization, as evidenced by lower gel viscosity (by up to 14%) and higher endpoint temperatures during pasting (by 2–4%). The inclusion of acorn flour in wheat dough notably affected its rheological properties, particularly by reducing dough extensibility (by up to 56%). However, farinographic parameters such as dough development time and stability time were extended (by 23–378% and 29–291%, respectively). High levels of acorn flour addition (>30%) resulted in bread with a dense, gummy, and less spongy crumb structure, accompanied by a reduction in loaf volume (by 40–52%). The maximal acceptable addition of acorn flour (30%) resulted in a two-fold increase in ash and fiber contents, along with decreases in carbohydrate and protein contents by 12% and 27%, respectively. These findings emphasize the need for careful formulation adjustments when incorporating acorn flour into wheat-based baked goods to balance technological efficiency and improved nutritional value.

## 1. Introduction

The behavior of flour during dough making and bread baking, along with the factors influencing this behavior, is referred to as the baking value of flour. It is a critical aspect of overall flour quality, determining the selection of technological process parameters, the need for recipe adjustments, including the potential use of improvers, and ultimately the quality of the final product [1]. Different types of flour exhibit variations in baking value due to factors such as chemical composition, amylolytic enzyme activity, starch granule size, and particle size distribution. These differences in baking value are reflected and visible at all stages of the technological process, i.e., from dough preparation and structure formation to baking. They also affect the characteristics of the final product, including bread volume, crumb elasticity and porosity, as well as sensory attributes [1,2]. Regardless of flour type, key elements of baking value include water absorption capacity, gas production during dough fermentation, starch behavior during pasting, rheological characteristics of starch gels, and the rheological properties of the dough. These factors determine changes in dough consistency and elasticity during mixing and shaping. The aforementioned parameters are typically evaluated through indirect methods based on instrumental measurements [3,4]; however, the most accurate assessment of the baking value of flour is obtained through direct methods, specifically laboratory baking tests. This approach involves dough preparation and bread baking under controlled conditions using small quantities of flour. Key conclusions about baking value are drawn from evaluations of dough yield, bread yield and volume, baking loss, as well as the porosity and structure of the bread crumb [5].

Acorn flour has been identified as a potential ingredient to enhance the nutritional value of bread and pastry products [6,7]. Its nutritional advantages include being gluten-free, high in dietary fiber and minerals, and rich in biologically active compounds such as natural antioxidants, including tocopherols and polyphenols [8,9,10,11]. However, alongside these advantages, acorn flour also presents disadvantages due to its lower baking value compared to traditional flours. Studies have demonstrated that incorporating acorn flour induces changes in the physical properties of dough and bread [6,12,13,14,15,16,17,18]. Acorn flour is characterized by a high water-absorption capacity, which positively impacts dough and bread yield; however, its inclusion negatively affects dough machinability and elasticity, resulting in dough that is less flexible and stretchable. Bread made with acorn flour exhibits lower density, reduced volume, decreased porosity, and increased hardness. Notably, existing research presents contradictory and ambiguous findings regarding the limiting effects of acorn flour on dough and bread properties. For example, in studies assessing the behavior of dough with acorn flour using a farinograph, Mousavi et al. [19] found that the addition of acorn flour (ranging from 10% to 50%) more than doubled dough development time (DDT) and dough stability time (DST) at the highest acorn flour content. In contrast, studies by Hruskova et al. [12] and Svec et al. [15] showed a two-fold extension of DST with the addition of just 10% acorn flour. Conversely, Rashid et al. [20] reported a reduction in both DDT and DST regardless of acorn flour content (from 15% to 45%). Different results were presented by Ajo [6], in which the addition of acorn flour (from 5% to 15%) shortened dough development time but extended dough stability time.

Many studies have explored the inclusion of acorn flour in various bakery products, including wheat bread, gluten-free bread, and regional baked goods (e.g., [6,8,13,14,15,16,17,18,21,22]). These studies used a range of acorn flour additions from 5% to 60%. Even at low acorn flour concentrations, a decrease in bread quality was observed, although the results—particularly regarding porosity—were inconsistent. For example, Gonzaga et al. [16] found a decrease in porosity and pore size in wheat bread with 10% and 15% acorn flour, and Skendi et al. [18] observed similar changes in gluten-free bread with 5%, 15%, and 25% acorn flour. In contrast, Purabdolah et al. [14] found increases in porosity and pore size, even with 10% acorn flour in wheat bread and Korus et al. [13] in gluten-free bread with 20%, 40%, and 60% acorn flour. Sensory evaluations also yielded inconsistent results, with some studies indicating consumer acceptance of acorn flour products [13,14,16,17], while others reported deterioration in sensory attributes such as taste, smell, or crumb texture [6,12,15].

It is important to note that there are few studies in which the optimal level of acorn flour addition to bread or pastry products is determined based on empirical data that assesses the impact of this addition on the baking properties of the base flour. To the best of our knowledge, no studies have conducted a comprehensive evaluation of dough properties, fermentation processes, and bread quality in relation to the use of different proportions of acorn flour. While previous research has explored individual aspects of acorn flour incorporation, such as its effect on dough rheology [12,15] or bread texture [13,14,17], these studies have not simultaneously addressed the full spectrum of technological properties, including fermentation behavior, dough development, and the resulting bread characteristics. A thorough understanding of how varying levels of acorn flour influence these factors is essential for optimizing the use of acorn flour in baking and for ensuring both the nutritional and sensory quality of the final product.

The purpose of this study was to evaluate the effect of acorn flour addition on the baking properties of wheat flour. Mixtures of wheat flour with varying proportions of acorn flour were analyzed for fermentographic, amylographic, farinographic, and extensographic properties, and a laboratory baking test was conducted. This study specifically examined the relationship between the level of acorn flour addition and changes in dough stability, development, and gas retention capacity. Additionally, specific parameters related to starch behavior during pasting, including gel viscosity and endpoint temperatures, were thoroughly examined.

## 2. Materials and Methods

### 2.1. Materials

**Figure 1:**
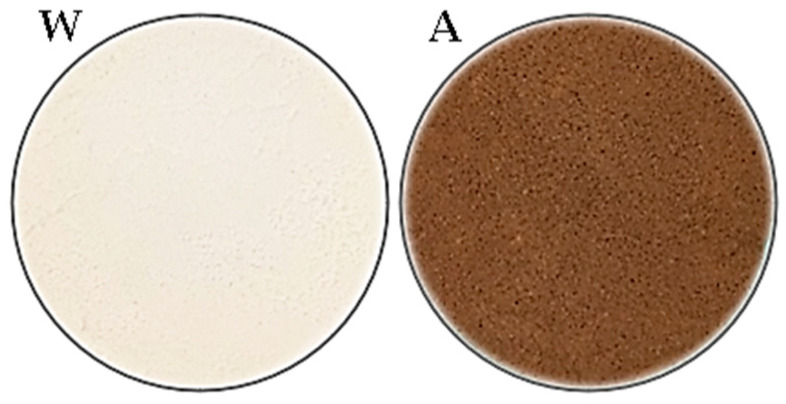
Images of wheat flour (W) and acorn flour (A) used in this study.

The commercial flours used in this study were acorn flour (Dary Natury, Koryciny, Poland) and white wheat flour, type 750 (Młyny Stoisław, Stoisław, Poland), as shown in Figure 1.

The characteristics of these flours are presented in Table 1. In the extensographic analysis and bread-baking experiments, in addition to the aforementioned flours, salt (Cenos, Września, Poland) and pressed baker’s yeast (Lesaffre Polska SA, Wołczyn, Poland) were also used.

The wheat–acorn flour samples were prepared by blending wheat flour with acorn flour at varying ratios (5–50%). The flours were thoroughly mixed to ensure a homogeneous distribution of acorn flour within the wheat flour. The samples used in this study were named systematically based on the percentage of wheat flour (W) and acorn flour (A), ranging from W100 + A0 to W0 + A100.

### 2.2. Analysis of Water Absorption

Water absorption was determined using a centrifugation-based method, following the standard AACC Method 56–20.01 [25], with some modifications. This study analyzed wheat flour (control), acorn flour, and mixtures of wheat flour and acorn flour. Acorn flour was substituted for wheat flour in proportions ranging from 5% to 50%. For mixtures containing up to 30% acorn flour, increments of 5% were used, while increments of 10% were applied for mixtures with acorn flour levels from 30% to 50%. A mass of 1.00 ± 0.01 g of flour was weighed into centrifuge tubes, followed by the addition of 20 cm^3^ of distilled water. The samples were homogenized for 1 min at a speed of 1000 rpm using a homogenizer. Subsequently, they were centrifuged for 15 min at 9000 rpm using an MPW-350 R centrifuge (MPW Med. Instruments, Warsaw, Poland). After centrifugation, the supernatant was carefully decanted, and the tubes were inverted onto filter paper to allow residual water to drain for 10 min. Water absorption capacity of the flour was calculated using the following formula:

water absorption (%) = ((a − b)∙100)/w(1)

where

a—mass of the test tube with wet sediment (g);
b—mass of the empty centrifuge tube (g);
w—mass of the weighed sample (g).

### 2.3. Analysis of Fermentographic Properties

Fermentographic analysis of the flour samples was carried out using a BZS SZ 2005 type laser fermentograph (Sadkiewicz Instruments, Bydgoszcz, Poland), following the methodology described by Sobczyk et al. [26]. The analysis included wheat flour (used as a control), acorn flour, and wheat–acorn flour mixtures. The substitution of wheat flour with acorn flour ranged from 5% to 50%, with increments of 5% for mixtures containing up to 30% acorn flour and increments of 10% for mixtures containing 30% to 50% acorn flour. The dough for fermentographic analysis was prepared with 140 g of flour, 2.5 g of yeast, 2 g of salt, and 80 cm^3^ of water. The ingredients were mixed for 7 min and then fermented in the fermentograph growth chamber at 35 °C. The fermentation time was set to 90 min, with measurements taken every 2 min. The following parameters were determined from the resulting fermentograms: the volume of retained gases (cm^3^); the volume of emitted gases (cm^3^); and the dough volume at the critical point (cm^3^).

### 2.4. Analysis of Amylographic Properties

Amylographic analysis of the flour samples was conducted according to the Polish Standard [27] using the Micro Visco-Amylo-Graph (Brabender^®^, GmbH & Co. KG, Duisburg, Germany). Wheat flour (as the control), acorn flour, and wheat–acorn flour mixtures were analyzed. The substitution of wheat flour with acorn flour ranged from 5% to 50%, with increments consistent with those used in the fermentographic analysis. A 15 g sample of flour or flour mixture was combined with 100 cm^3^ of water and transferred quantitatively into the measuring bowl. The suspension was immediately measured, and the heating was initiated at 30 °C and increased to 92 °C (held constant for 1 min) at a rate of 7.5 °C/min. The mixing speed was set at 250 rpm. Based on the resulting amylograms, the following parameters were determined: the temperature and viscosity at the start of starch pasting; and the temperature and viscosity at the end of starch pasting.

### 2.5. Analysis of Farinographic Properties

Farinographic analysis of the flour samples was conducted using a Brabender Farinograph^®^ (Brabender^®^, GmbH & Co. KG, Duisburg, Germany), following the guidelines outlined in the Polish Standard [28]. Wheat flour (used as the control), acorn flour, and wheat–acorn flour mixtures were analyzed. The substitution of wheat flour with acorn flour ranged from 5% to 25%, with increments of 5%. Farinographic analysis did not yield reliable results for flour mixtures containing more than 25% acorn flour. A sample size of 300 g (±0.5 g) was used for the analysis. Water absorption, as determined in Section 2.2, was applied to obtain a dough. The mixing time was set to 15 min at a speed of 63 rpm. The results were evaluated in accordance with the Brabender^®^/ICC/BIPEA standard, providing the following parameters: dough consistency; dough development time; dough stability time; degree of softening (measured 10 min after the start of mixing); and the quality number.

### 2.6. Analysis of Extensographic Properties

Extensographic analysis of the flour samples was performed using a Brabender Extensograph^®^-E (Brabender^®^, GmbH & Co. KG, Duisburg, Germany), according to the Polish Standard [29]. Wheat dough (used as the control) and wheat–acorn doughs were analyzed. The substitution of wheat flour with acorn flour ranged from 5% to 25%, with increments of 5%. Dough samples were prepared using a Farinograph kneader with flour, water, and 2% salt. The water addition was adjusted to achieve a dough consistency of 500 ± 20 FU. Portions of 150 ± 0.5 g of dough were weighed and shaped using the extensographic former. The formed dough cylinders were placed in cassettes and fermented in the apparatus chamber at 30 °C. Measurements (stretching) were taken after 30, 60, and 90 min of fermentation, determining the following dough parameters: total energy required for stretching; resistance to extension; maximum resistance at the breaking point; extensibility; and the ratio number.

### 2.7. Bread Making

Bread baking was carried out using the straight-dough method with 1-h fermentation. As a control sample, a white wheat bread was prepared. For the acorn bread variants, wheat flour was substituted with acorn flour at levels ranging from 10% to 50%, in increments of 10%.

In the straight-dough method, 6 g of yeast and 4 g of salt were added per 200 g of flour. The amount of water added varied and was adjusted based on the water absorption properties of the flour samples, as determined in Section 2.2. The yeast and salt were dissolved in a portion of water. Then the flour, salt, and yeast solutions, and the remaining water were mixed using a universal mixer equipped with a kneading hook (JAZ S.R.O., Nové Mesto nad Váhom, Slovakia) for 5 min. After mixing, the dough was placed in a growth chamber (Wachtel, Hilden, Germany) and fermented at 35 °C with 85% relative humidity for 60 min. For the wheat flour variant (without acorn flour addition), the dough was punctured after 30 min of fermentation. Following fermentation, 300 g bites were shaped and placed into baking forms with dimensions of 17 cm × 8.5 cm × 7 cm (length × width × height). The dough was then subjected to secondary fermentation at 35 °C and 85% relative humidity for approximately 30 min to achieve optimal volume (the dough adhered to the form walls, the top surface became nearly flat, and the dough exhibited no resistance when pressed). The bread was then baked in a modular bakery and confectionery oven with a growth chamber (Quail Mini Piccolo 4 bakery oven; Wachtel, Germany) at 230 °C for 30 min. Each bread variant was prepared in three independent replicates. After baking, bread samples were cooled, and bread volume was analyzed 24 h post-baking.

### 2.8. Analysis of Technological Properties and Nutritional Value of Bread

The dough and bread yields, as well as oven and total losses, were quantified using mathematical calculations based on the methodology outlined by Mohammadi et al. [30]. Dough yield was determined as the ratio of the dough mass after fermentation to the initial mass of flour used for dough preparation. Bread yield was calculated using the masses of the dough portions, baked loaves, and the dough yield. Oven losses were evaluated as the difference between the mass of the dough portions and the warm baked loaves, while total losses were calculated as the difference between the masses of the dough portions and the cooled bread loaves.

Bread volume was measured automatically using a TexVol BVM 6630 device (Perten Instruments, Hägersten, Sweden). The entire loaf was analyzed by placing it on a rotating platform inside the machine. Measurements were performed using laser topography technology, where a laser sensor mounted on a movable arm scanned the loaves in a semicircular trajectory.

The hardness of the bread crumb was assessed using a TA.HDplus texture analyzer (Stable Microsystem Ltd., Godalming, UK). The parameter was defined as the force required to compress the crumb. Cubes of crumb with dimensions of 25 mm per side were cut from the central part of the loaf and subjected to compression using a compression platen (100 mm in diameter) as the compressive element, operating at a test speed of 2 mm/s. The compression distance was set at 50% deformation of the crumb. Ten measurements were conducted for each bread variant, and the results were expressed as the maximum force (N) required to compress the sample.

The nutritional value of the breads was evaluated by mathematically calculating the content of carbohydrates, protein, lipids, ash, and dietary fiber based on the composition of the raw materials (analytically determined nutrient content in acorn flour and wheat flour, presented in Table 1). The calculations accounted for mass losses during baking (total baking loss). Results were expressed as grams of each nutrient per 100 g of the product.

### 2.9. Statistical Analysis

Statistical evaluation of the results was performed using analysis of variance (ANOVA), followed by Duncan’s test. All calculations were carried out using Statistica 13.3 PL software (StatSoft, Kraków, Poland) at a significance level of *p* ≤ 0.05.

## 3. Results and Discussion

### 3.1. Effect of Acorn Flour Addition on Fermentographic Properties of Wheat Flour

Acorn flour differentiates the fermentographic and amylographic characteristics of wheat flour. Even a small addition of acorn flour (5%) influenced such parameters as the volume of gases released during fermentation (Table 2) and the starch gelatinization temperature (Table 3).

The addition of acorn flour to wheat flour influenced both the volume of gases released during fermentation and the ratio between released and retained gases. As the proportion of acorn flour increased, the volume of released gases increased (up to 140% in a sample containing 30% acorn flour), while the volume of retained gases decreased (up to 92% in a sample with 50% acorn flour). In samples with an acorn flour content exceeding 25%, the dough volume reached a critical point earlier, indicating the moment at which the dough achieves optimal fluffiness. The time to this critical point decreased from 32.67 min (control) to 18.67 min (data not presented).

In terms of starch pasting, this process is particularly crucial in gluten-free doughs or doughs incorporating gluten-free ingredients as it contributes to the entrapment and retention of gas bubbles within the starch matrix, which directly affects the final bread volume and porosity [31]. Accordingly, amylographic analysis is integral to evaluating fermentation properties. From the technological point of view, the most notable effect of acorn flour addition on the amylographic properties of wheat flour was a reduction in the maximum viscosity of the starch gels. This decrease became statistically significant at acorn flour levels of 30% or higher. Correia et al. [32] previously reported that acorn starch exhibits limited swelling capacity, which influences the gelatinization process and the properties of the resulting gels. Similarly, Beltrão Martins et al. [8] found that samples containing acorn flour demonstrated lower viscosity compared to the mixture of rice, potato, and buckwheat flours. The observed differences in starch gelatinization properties may be attributed to acorn starch characteristics, especially shape and size, and variations in amylose content [33]. The relationship between amylose and fat content among different flours may also be important [34]. Acorn starch granules take on various shapes—spherical, elliptical, and irregular—with a wide range of sizes (diameters from 3.3 μm to 126.2 μm) and with pores on the surface. The share of amylose is also varied (from 19.5% to 59.4%) [35,36]. The morphology of granules and the share of amorphous and crystalline regions resulting from molecular rearrangements of amylose and amylopectin fractions translate into starch’s ability to interact with water and transition from an ordered to a disordered state, which is a condition for the proper and complete course of the gelatinization process [33,37]. As noted by Huang et al. [38], amylose–lipid complexes may form, which can reduce the swelling capacity and water solubility of starch granules. Furthermore, the addition of acorn flour to wheat flour increased the final gelatinization temperature; however, this increase in the end-point temperature of starch pasting was not statistically significant (*p* > 0.05) across different acorn flour concentrations. On average, the temperature increased by 3.5% in samples containing acorn flour compared to the control sample (wheat flour). This result was consistent with findings presented by Beltrão Martins et al. [8].

### 3.2. Effect of Acorn Flour Addition on Farinographic Parameters of Wheat Flour

**Figure 2:**
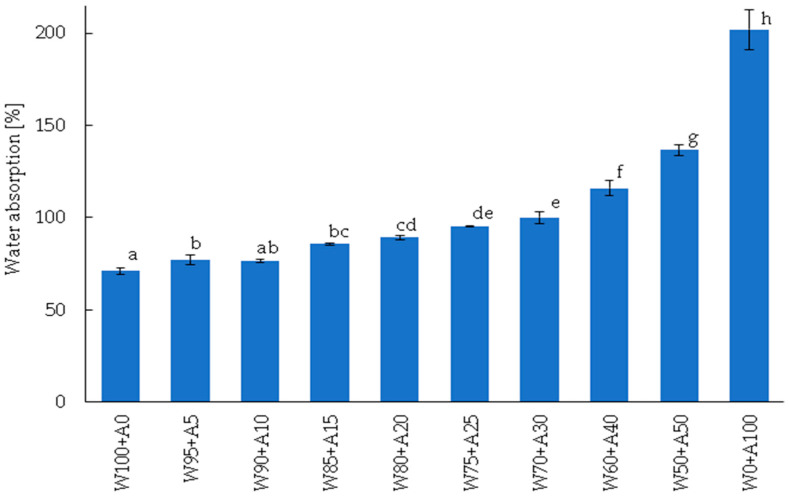
Water absorption (mean ± standard deviation, *n* = 3) of wheat–acorn flour samples. Abbreviations: W—wheat flour; A—acorn flour; 0, 5, … 100—indicates the percentage of wheat/acorn flour in the total flour content of the sample; ^a, b, c, …^—different letters above the bars (representing mean values) denote statistically significant differences at *p* ≤ 0.05, as determined by Duncan’s test.

Evaluating the baking quality of flour and its suitability for mechanical processing through farinographic analysis provides insights under conditions that closely resemble industrial production, making it a more practical approach than solely assessing protein quantity and quality [39]. The addition of acorn flour to wheat flour affects both water absorption (Figure 2) and the rheological properties of the resulting dough, as measured by the Brabender^®^ Farinograph (Table 4). It is important to note that reliable farinographic evaluation was not feasible for mixed flour samples with acorn flour proportions exceeding 25%. The results obtained under these conditions were inconsistent, leading to discrepancies between the Farinograph^®^ readings and the observed structure and consistency of the dough. As suggested by Saleh et al. [40], the relationship between flow properties, particle size, and flour behavior during mixing in the Farinograph^®^ mixer indicates that the observed difficulties in analyzing acorn flour mixtures with high substitution levels may be attributed to the physical properties of acorn flour, particularly its poor flow characteristics. The lower flowability of acorn flour compared to wheat flour may be attributed to the different compositions of the two flours (Table 1).

Acorn flour exhibits significantly higher water absorption compared to wheat flour, with an absorption capacity nearly three times greater. This difference arises from variations in the chemical composition and structural characteristics of the two flours, particularly the high dietary fiber content in acorn flour (Table 1), differences in granulation (Figure 1), and the size and degree of degradation of starch granules [19]. The elevated water absorption capacity of acorn flour increased the water absorption of mixed flour samples. A direct correlation was observed: the higher the proportion of acorn flour in the mixture, the greater the water absorption. The increase ranged from 8% in samples with 5% acorn flour to 92% in those with 50%. According to Cacak-Pietrzak et al. [41], higher water absorption directly enhances bread yield and may affect its textural attributes.

The addition of acorn flour also extended the dough development time and dough stability time. For wheat flour alone, the dough development time was 4.68 min. This time increased with higher proportions of acorn flour: a 5% addition extended the development time by 21%; a 20% addition by 166%; and a 25% addition by 377%. Similarly, the dough stability time increased significantly, from 9.2 min to 36.1 min (a 292% increase) for the sample with 25% acorn flour. These findings are consistent with those of Mousavi et al. [19], who also observed an extension of dough development and stability times, though the reported increases were lower, with maximum extensions of 109% and 153%, respectively, for samples with a 50% acorn flour substitution. In contrast, Švec et al. [15] found that a 5% addition of acorn flour increased dough development time by 209%, while dough stability time decreased by 59%. Rashid et al. [20] observed reductions in both dough development and stability times with increasing acorn flour proportions, whereas Ajo [6] reported shortened dough development time but extended dough stability time with acorn flour addition.

Acorn flour, being gluten-free, might theoretically weaken the gluten network, thereby reducing both dough development and stability times; however, the present study observed extensions of these parameters. This unexpected behavior can be attributed to the chemical composition of acorn flour, particularly its high dietary fiber content (Table 1). Dietary fiber has a strong water-binding capacity and can interact with gluten to form fiber–protein aggregates. These molecular interactions may partially dehydrate the gluten network and induce conformational changes in gluten proteins. Previous studies [42,43,44,45] have confirmed that the addition of dietary fiber can slow hydration during mixing due to differences in the water-binding capacity of non-starch polysaccharides (e.g., arabinoxylans, hemicelluloses, and lignins), resulting in extended farinographic parameters. Furthermore, the high fat content in acorn flour (Table 1) could contribute to these changes. Fat can interact with gluten proteins and affect their hydration: the water absorption capacity of glutenin decreases in the presence of lipids, while gliadins increase [46].

### 3.3. Effect of Acorn Flour Addition on Extensographic Parameters of Wheat Flour

Extensographic analysis revealed changes in the rheological properties of wheat dough, specifically in its extensibility and resistance to extension, as the proportion of acorn flour in the sample increased (Table 5).

The addition of acorn flour to wheat dough at levels between 5% and 15% increased the dough’s resistance to stretching. For a fermentation time of 30 min, the highest resistance was observed in the sample containing 10% acorn flour, while for fermentation times of 60 and 90 min, the maximum resistance was recorded for the sample with 5% acorn flour. Further substitution of wheat flour with acorn flour led to a decrease in resistance compared to the control sample. The limiting effect of acorn flour on the extensibility of wheat dough, as well as the initial increase in resistance to stretching with moderate acorn flour additions, was also observed by Mousavi et al. [19]. Similarly, studies conducted by Gonzaga et al. [16] demonstrated that the addition of 10% and 15% acorn flour increased the energy required for stretching, enhanced the dough’s extensibility, and improved its resistance to stretching. As noted by Dapčević Hadnađev et al. [47], the energy parameter in extensographic evaluation serves as an indicator of flour strength. Stronger flours require greater energy to stretch the dough. Mirza Alizadeh et al. [48] further reported that the energy required for stretching is correlated with the dough’s gas retention capacity, the baked bread’s volume, and its handling characteristics during shaping; the findings of the present study align with these observations. Specifically, the inclusion of acorn flour reduced the energy required to stretch the dough (Table 5), decreased the volume of retained gases (Table 2), and produced bread with smaller volumes in laboratory baking tests (Table 6). One critical factor influencing extensographic parameters is the quantity and quality of gluten in the flour, particularly the ratio of gliadin to glutenin [47]. Since acorn flour is gluten-free, this likely explains the observed unfavorable changes in dough extensibility when acorn flour is incorporated [49].

### 3.4. Effect of Acorn Flour Addition on Technological Properties of Wheat Dough and Bread

As shown in Table 6, the addition of acorn flour significantly alters the technological properties of dough and bread samples. Notably, as the proportion of acorn flour increased, both the dough and bread yield showed a statistically significant increase, while the loaf volume decreased. Oven losses and total baking losses remained largely unaffected for acorn flour proportions of up to 40%. These findings are consistent with the results reported by Beltrão Martins et al. [8], who similarly observed no significant changes in baking losses for gluten-free bread containing acorn flour.

The addition of acorn flour to wheat bread, baked using the direct method, significantly increased dough yield, with gains ranging from 5% to 30% for samples containing 10% and 50% acorn flour, respectively, compared to the control sample. Statistically significant increases in dough yield were observed between successive samples, with each 10% increment in acorn flour addition resulting in an average increase of 5.35%. As dough yield increased, bread yield also rose correspondingly. The inclusion of acorn flour increased bread yield by up to 22% in samples with 50% acorn flour. Statistically significant increases in bread yield were observed for every 20% increase in acorn flour incorporation. These increases in dough and bread yield are directly attributable to the high water absorption capacity of acorn flour, as previously reported by Mousavi et al. [19]. In contrast, the volume of bread exhibited an inverse trend. An increase in the proportion of acorn flour led to a statistically significant (*p* ≤ 0.05) decrease in bread volume, with each 10% increment in acorn flour causing an average reduction of 37%. Similar trends were reported by Park et al. [17] and Hrušková et al. [12] for wheat breads, as well as by Korus [13] and Skendi et al. [18] for gluten-free breads. Hrušková et al. [12] demonstrated that the addition of more than 5% acorn flour to wheat flour resulted in decreased bread volume, while Park et al. [17] observed a 28% reduction in the volume of wheat bread containing 25% acorn flour. Studies by Korus et al. [13] and Skendi et al. [18] indicated that the volume of gluten-free bread decreased when the proportion of acorn flour exceeded 15–20%.

The addition of acorn flour increased the crumb hardness (Table 6). The higher the proportions of acorn flour in the sample, the higher the value of the force needed to compress the bread crumb. For wheat bread, the crumb hardness was 20.15 N. This hardness increased with higher proportions of acorn flour: a 10% addition increased the hardness by 21%; a 30% addition by 45%; and a 50% addition by 52%. The increase in bread crumb hardness could be related to the high content of dietary fiber in acorn flour (Table 1), which translates into gluten hydration and dough behavior during mixing and fermentation as well as the formation of an elastic, porous crumb structure. These findings are consistent with those of Mousavi et al. [19], who also observed an increase in crumb hardness, though the reported increases were higher, with an increase of 90% for samples of Iranian toast bread with a 30% acorn flour substitution. Park et al. [17] and Purabdolah et al. [14] confirmed, similarly to the present study, that the addition of acorn flour affected the hardness of bread but did not influence other textural features, including elasticity or cohesiveness.

**Figure 3:**
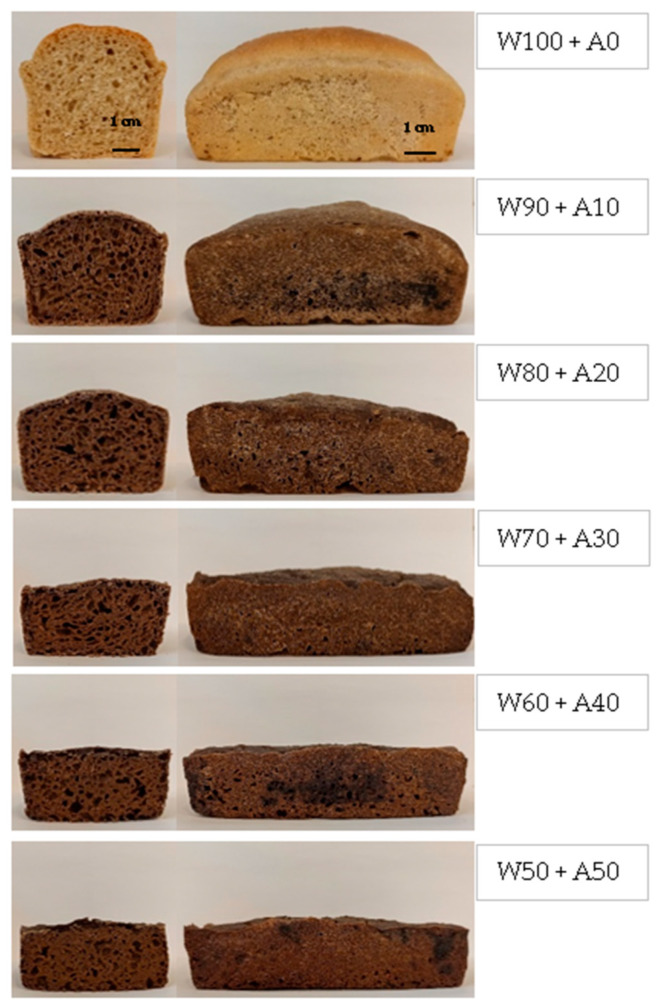
Cross-sectional and side views of wheat–acorn bread samples. Explanations: W—wheat flour; A—acorn flour; 0, 10, … 100—indicates the percentage of wheat/acorn flour in the total flour content of the sample.

Visual differences in bread color, volume, and crumb structure are apparent in the loaves (Figure 3). The incorporation of higher proportions of acorn flour results in bread with a dense, less-spongy, and gummy crumb with reduced volume. Crumb porosity also deteriorates; as the acorn flour content increases, the distribution and size of pores become more irregular. Since acorn flour is gluten-free, its lack of gluten negatively impacts bread porosity and volume, as confirmed by numerous studies (e.g., [12,17,50,51,52,53]). It was also observed that even at the lowest level of acorn flour addition (10%), a noticeable darkening of the bread color occurred; however, as the proportion of acorn flour increased, further color changes were minimal. This can be explained by the distinctly darker color of the acorn flour used in this study, as shown in Figure 1.

The effect of acorn flour on the nutritional value of wheat bread was presented using the example of bread with 30% acorn flour (Table 7). The addition of acorn flour to bread affected its nutritional value and the content of basic nutrients. The differences concerned all the assessed ingredients: carbohydrates, protein, lipids, ash, and fiber. Acorn flour caused a decrease in the content of carbohydrates (by 12%) and protein (by 27%) and an increase in the content of lipids, ash, and fiber (by 18%, 71%, and 111%, respectively). The effect of adding acorn flour on the nutritional value was also assessed in studies by other authors in breads based on flours of various origins: wheat and gluten-free [8,12,13,15]. Hrŭskova et al. [12] and Švec et al. [15] noted an increase in the fiber content in wheat bread with the addition of acorn flour, even at the level of 5–10%. A decrease in the protein content and an increase in the ash and fiber contents in wheat bread (bread in the form of flatbreads) was shown by Ajo [6]. Korus et al. [13] noted the effect of acorn flour on the increase in the fat and dietary fiber content in gluten-free bread. The authors also indicated an increase in protein content, which is different compared to the results obtained in this study. These differences can probably be explained by the different compositions of the base flours.

In addition to the content of basic nutrients, the effect of acorn flour addition on the fatty acid profile of bread will also be significant. The fat of acorn flour is dominated by unsaturated fatty acids, and the main fatty acids are oleic acid (up to 61% of the sum of all fatty acids) and linoleic acid (up to 16%) [9]. It is worth emphasizing that the fatty acid profile of acorn flour is different from the fatty acid profiles of cereal flours, in which linoleic acid dominates (up to 61.00% of the sum of all fatty acids in wheat flour) [54,55]. Higher fat content in acorn flour (Table 1) will affect the fatty acid profile of bread, which may affect the course of fat oxidation in bread during baking and storage. Linoleic acid is at least 20 times more susceptible to oxidation than oleic acid [56]; therefore, it can be assumed that in bread with the addition of acorn flour, the formation of oxidation products will be slower. It is also worth mentioning that the fatty acid profile is also important for nutritional reasons. It is indicated that the consumption of oleic acid may contribute to supporting the treatment and prevention of circulatory system diseases, metabolic disorders, and thrombotic dysfunctions [57].

Acorn flour is also a raw material enriching the composition of bread with minerals, due to the higher ash content (Table 1). Among the minerals in acorn flour, calcium and iron dominate but it also contains significant amounts of magnesium, copper, and manganese [58,59]. Acorn flour can, therefore, be a source of calcium and iron in bread with its participation and increase the amount of these ingredients in the diet.

## 4. Conclusions

The conducted experiments demonstrate that acorn flour significantly impacts the baking properties of wheat flour, affecting its fermentographic, amylographic, farinographic, and extensographic characteristics, as well as determining the technological and physical properties of wheat dough and bread. The findings highlight the necessity of optimizing dough preparation and bread-baking parameters when incorporating acorn flour compared to traditional methods. Dough containing acorn flour exhibited extended development time and stability in farinographic assessments, suggesting a need for prolonged mixing. Conversely, the highest tensile resistance values displayed at the shortest fermentation times in the extensographic evaluations, coupled with a reduced time to achieve maximum volume in fermentographic assessments, indicate a potential requirement to shorten the fermentation time.

Laboratory baking tests revealed that acorn flour is unsuitable as a standalone raw material for bakery production. However, due to its high nutritional value, it can be effectively utilized as a nutritional enrichment additive. The results indicate that incorporating acorn flour into wheat bread formulations should not exceed 30%, as higher levels negatively impact gas retention during fermentation and alter the viscosity of the starch gel during the final stages of pasting. These effects compromise key quality parameters of the bread, particularly the loaf volume. Future research could focus on the potential of blending acorn flour with wheat flour possessing strong gluten-forming properties. High-gluten wheat flour can counteract the weakening effects of gluten dilution, ensuring the maintenance of dough elasticity, gas retention, and overall bread volume.

## Figures and Tables

**Table 1:** Quality properties of wheat and acorn flours.

Quality Parameters		Wheat Flour (W)	Acorn Flour (A)
Color parameters	L*	92.42	50.92
	a*	0.21	6.81
	b*	7.80	23.07
Chemical composition	Water content (g/100 g)	13.5	11.6
	Carbohydrate content (g/100 g)	68.7	61.8
	Protein content (g/100 g)	12.6	4.1
	Fat content (g/100 g)	1.8	3.7
	Total dietary fiber content (g/100 g)	2.9	16.8
	Ash content (g/100 g)	0.5	2.0
Technological value	Wet gluten content (%)	31	n.d.
	Gluten spreadability (mm)	2	n.d.
	Zeleny sedimentation index (cm^3^)	36	n.d.
	Falling number (s)	374	n.d.
	Acidity (cm^3^ sodium hydroxide/100 g)	5.56	n.d.

n.d.—not determined. Color parameters were measured using Hunter ColorFlex EZ (HunterLab, Reston, VA, USA). Contents of water, protein, fat, fiber, and ash were determined using standard AOAC Official Methods [23] (methods 925.10, 960.52A, 923.05, 920.86, and 923.03, respectively). The carbohydrate content was calculated by the difference between 100 g and the content of determined compounds. The wet gluten content and its spreadability, Zeleny’s sedimentation index, falling number, and acidity of wheat flour were determined using ICC Standard Methods [24] (methods 150, 116/1, 107/1, and 145, respectively).

**Table 2:** Fermentographic properties of wheat and wheat–acorn flour samples.

Sample Type	Retained Gases (cm^3^)	Released Gases (cm^3^)	Volume at Critical Point (cm^3^)
W100 + A0	177.0 ± 3.6 ^a^	146.3 ± 7.6 ^a^	59.7 ± 6.7 ^a^
W95 + A5	175.0 ± 18.5 ^ab^	201.3 ± 25.0 ^b^	58.7 ± 5.5 ^a^
W90 + A10	172.3 ± 9.7 ^ab^	256.3 ± 29.7 ^c^	58.0 ± 1.5 ^a^
W85 + A15	163.0 ± 5.6 ^b^	275.3 ± 14.6 ^cd^	63.0 ± 7.0 ^a^
W80 + A20	113.3 ± 2.5 ^c^	279.7 ± 35.6 ^cd^	53.3 ± 5.5 ^a^
W75 + A25	86.3 ± 6.5 ^d^	339.6 ± 27.1 ^ef^	42.0 ± 4.4 ^b^
W70 + A30	64.0 ± 4.0 ^e^	351.3 ± 29.6 ^f^	37.0 ± 2.0 ^bc^
W60 + A40	50.0 ± 1.0 ^f^	309.3 ± 9.1 ^de^	31.7 ± 1.5 ^c^
W50 + A50	13.3 ± 1.5 ^g^	167.7 ± 5.9 ^ab^	24.7 ± 1.1 ^d^
W0 + A100	66.3 ± 2.5 ^e^	15.7 ± 3.2 ^g^	57.0 ± 4.4 ^a^

The results are the mean ± standard deviation (n = 3); W—wheat flour; A—acorn flour; 0, 5, … 100—indicates the percentage of wheat/acorn flour in the total flour content of the sample; ^a, b, c, …^—mean values within a column marked with different superscript letters indicate statistically significant differences at *p* ≤ 0.05, as determined by Duncan’s test.

**Table 3:** Amylographic properties of wheat and wheat–acorn flour samples.

Sample Type	Start of Starch Pasting		End of Starch Pasting	
	Temperature (°C)	Viscosity (BU)	Temperature (°C)	Viscosity (BU)
W100 + A0	58.6 ± 0.2 ^a^	17.7 ± 0.6 ^ab^	85.3 ± 0.6 ^a^	744.7 ± 6.0 ^a^
W95 + A5	58.6 ± 0.2 ^a^	17.3 ± 0.6 ^ab^	86.7 ± 0.1 ^b^	730.7 ± 8.5 ^a^
W90 + A10	59.6 ± 0.7 ^ab^	17.3 ± 0.6 ^ab^	88.6 ± 1.1 ^c^	726.3 ± 16.0 ^ab^
W85 + A15	60.7 ± 2.2 ^b^	16.3 ± 2.1 ^b^	88.2 ± 0.1 ^c^	722.0 ± 12.0 ^ab^
W80 + A20	59.3 ± 0.2 ^ab^	18.3 ± 0.6 ^a^	88.6 ± 0.8 ^c^	720.0 ± 5.3 ^ab^
W75 + A25	60.5 ± 0.5 ^b^	17.7 ± 0.6 ^ab^	88.8 ± 0.6 ^c^	719.3 ± 17.0 ^ab^
W70 + A30	60.7 ± 0.4 ^b^	16.7 ± 0.6 ^ab^	88.5 ± 0.1 ^c^	694.0 ± 6.2 ^b^
W60 + A40	60.5 ± 0.6 ^b^	16.3 ± 0.6 ^b^	88.3 ± 0.4 ^c^	661.0 ± 7.8 ^c^
W50 + A50	61.3 ± 0.5 ^b^	17.3 ± 1.5 ^ab^	88.5 ± 0.5 ^c^	638.7 ± 3.1 ^c^
W0 + A100	69.7 ± 1.2 ^c^	16.7 ± 0.6 ^ab^	99.9 ± 0.1 ^d^	531.0 ± 4.3 ^d^

The results are the mean ± standard deviation (*n* = 3); W—wheat flour; A—acorn flour; 0, 10, … 100—indicates the percentage of wheat/acorn flour in the total flour content of the sample; ^a, b, c, …^—mean values within a column marked with different superscript letters indicate statistically significant differences at *p* ≤ 0.05, as determined by Duncan’s test.

**Table 4:** Farinographic parameters of wheat and wheat–acorn flour samples.

Sample Type	Consistency (FU)	Dough Development Time (min)	Dough Stability Time (min)	Degree of Softening (FU)	Quality Number
W100 + A0	499.3 ± 10.5 ^ab^	4.7 ± 0.1 ^a^	9.2 ± 0.2 ^a^	34.3 ± 1.1 ^a^	94.3 ± 2.1 ^a^
W95 + A5	483.0 ± 4.6 ^a, b^	5.7 ± 0.2 ^ab^	11.9 ± 0.6 ^b^	21.3 ± 2.5 ^b^	113.3 ± 6.5 ^ab^
W90 + A10	491.7 ± 3.2 ^ab^	7.0 ± 0.4 ^b^	13.5 ± 0.3 ^c^	13.3 ± 1.5 ^c^	123.0 ±1.0 ^b^
W85 + A15	513.0 ± 24.5 ^c^	7.5 ± 0.4 ^b^	10.6 ± 0.6 ^ab^	12.3 ± 2.5 ^c^	119.7 ± 4.5 ^b^
W80 + A20	494.7 ± 23.7 ^ab^	12.5 ± 1.0 ^c^	29.9 ± 2.4 ^d^	5.7 ± 1.5 ^d^	236.3 ± 32.6 ^c^
W75 + A25	476.0 ± 47.5 ^a^	22.4 ± 3.7 ^d^	36.1 ± 0.2 ^e^	34.3 ± 10.2 ^a^	319.7 ± 17.6 ^d^

The results are the mean ± standard deviation (*n* = 3); W—wheat flour; A—acorn flour; 0, 5, … 100—indicates the percentage of wheat/acorn flour in the total flour content of the sample; ^a, b, c, …^—mean values within a column marked with different superscript letters indicate statistically significant differences at *p* ≤ 0.05, as determined by Duncan’s test.

**Table 5:** Extensographic parameters of wheat and wheat–acorn dough samples.

Parameter/Time		W100 + A0	W95 + A5	W90 + A10	W85 + A15	W80 + A20	W75 + A25
Total energy (cm^2^)	30 min	122.3 ± 11.4 ^a^	84.0 ± 4.6 ^b^	74.7 ± 7.6 ^bc^	63.7 ± 2.1 ^cd^	55.7 ± 4.5 ^d^	39.0 ± 3.0 ^e^
	60 min	185.0 ± 8.7 ^a^	132.0 ± 5.0 ^b^	82.3 ± 7.0 ^c^	69.7 ± 8.5 ^d^	63.3 ± 4.9 ^d^	43.0 ± 2.6 ^e^
	90 min	154.0 ± 3.5 ^a^	121.3 ± 20.3 ^b^	70.0 ± 4.0 ^c^	69.3 ± 7.6 ^c^	57.3 ± 2.5 ^cd^	44.3 ± 3.2 ^d^
Resistance of dough to extension (EU)	30 min	573.0 ± 64.5 ^a^	651.3 ± 26.1 ^ab^	773.0 ± 54.1 ^c^	707.7 ± 26.0 ^bc^	587.0 ± 60.3 ^a^	350.3 ± 35.4 ^d^
	60 min	942.3 ± 4.2 ^a^	1105.3 ± 15.3 ^b^	1047.7 ± 27.6 ^b^	776.0 ± 40.0 ^c^	529.3 ± 84.2 ^d^	445.3 ± 44.0 ^e^
	90 min	990.0 ± 5.3 ^a^	1166.0 ± 10.8 ^b^	618.0 ± 82.5 ^c^	609.0 ± 88.9 ^c^	401.7 ± 23.6 ^d^	329.0 ± 98.6 ^d^
Extensibility (mm)	30 min	128.7 ± 0.6 ^a^	95.7 ± 1.1 ^b^	73.7 ± 3.1 ^c^	67.3 ± 3.2 ^d^	59.3 ± 2.3 ^e^	59.33 ± 3.5 ^e^
	60 min	125.0 ± 3.6 ^a^	93.3 ± 4.2 ^b^	63.7 ± 3.2 ^c^	55.3 ± 4.7 ^d^	56.3 ± 1.5 ^d^	56.0 ± 3.0 ^d^
	90 min	104.7 ± 2.1 ^a^	88.7 ± 3.5 ^b^	55.3 ± 0.6 ^c^	56.7 ± 1.5 ^c^	53.7 ± 1.5 ^c^	56.3 ± 3.1 ^c^
Maximum resistance at breaking point (EU)	30 min	713.7 ± 70.1 ^ab^	665.3 ± 27.4 ^a^	776.3 ± 55.0 ^b^	762.0 ± 22.3 ^ab^	742.7 ± 47.7 ^ab^	442.0 ± 72.2 ^c^
	60 min	1137.7 ± 22.7 ^a^	1164.7 ± 5.5 ^a^	1257.0 ± 19.7 ^a^	1103.0 ± 68.1 ^a^	885.3 ± 19.2 ^b^	560.7 ± 40.8 ^c^
	90 min	1224.7 ± 6.4 ^a^	1237.7 ± 13.9 ^a^	1122.0 ± 95.4 ^a^	1064.3 ± 77.7 ^a^	796.0 ± 20.9 ^b^	564.0 ± 26.5 ^c^
Ratio number	30 min	4.4 ± 0.5 ^a^	6.8 ± 0.2 ^b^	10.5 ± 0.2 ^c^	10.5 ± 0.9 ^c^	9.9 ± 0.8 ^c^	5.9 ± 0.7 ^d^
	60 min	7.5 ± 0.1 ^a^	11.9 ± 0.7 ^b^	16.5 ± 0.52 ^c^	14.1 ± 0.9 ^d^	9.4 ± 1.7 ^e^	8.0 ± 1.0 ^ae^
	90 min	9.5 ± 0.1 ^a^	13.1 ± 0.7 ^b^	11.1 ± 1.4 ^a^	10.7 ± 1.3 ^a^	7.3 ± 0.5 ^c^	5.8 ± 0.6 ^c^

The results are the mean ± standard deviation (*n* = 3); W—wheat flour; A—acorn flour; 0, 5, … 100—indicates the percentage of wheat/acorn flour in the total flour content of the sample; ^a, b, c, …^—mean values within a line marked with different superscript letters indicate statistically significant differences at *p* ≤ 0.05, as determined by Duncan’s test.

**Table 6:** Technological properties of wheat and wheat–acorn dough and bread samples.

Bread Samples	Dough Yield (%)	Bread Yield (%)	Bread Volume (cm^3^)	Oven Losses (%)	Total Losses (%)	Crumb Hardness (N)
W100 + A0	181.1 ± 1.0 ^a^	152.7 ± 2.8 ^a^	805.0 ± 7.1 ^a^	7.2 ± 1.1 ^a^	15.7 ± 1.1 ^a^	20.15 ± 0.56 ^a^
W90 + A10	189.7 ± 2.1 ^b^	158.0 ± 4.3 ^a^	668.0 ± 19.5 ^b^	9.5 ± 1.8 ^abc^	16.7 ± 1.8 ^a^	24.32 ± 1.41 ^b^
W80 + A20	200.4 ± 1.5 ^c^	168.6 ± 4.4 ^b^	571.8 ± 3.3 ^c^	7.7 ± 1.1 ^ab^	15.8 ± 2.5 ^a^	27.75 ± 0.67 ^c^
W70 + A30	208.7 ± 0.8 ^d^	174.0 ± 1.2 ^b^	484.9 ± 6.6 ^d^	10.3 ± 0.4 ^c^	16.6 ± 0.3 ^a^	29.15 ± 1.12 ^cd^
W60 + A40	215.6 ± 0.3 ^e^	182.1 ± 1.3 ^c^	435.9 ± 4.3 ^e^	8.1 ± 0.3 ^abc^	15.5 ± 0.5 ^a^	29.24 ± 1.02 ^cd^
W50 + A50	234.9 ± 1.8 ^f^	186.2 ± 5.1 ^c^	387.5 ± 15.1 ^f^	10.0 ± 1.8 ^bc^	20.7 ± 2.6 ^b^	30.57 ± 1.04 ^d^

The results are the mean ± standard deviation (*n* = 3); W—wheat flour; A—acorn flour; 0, 10, … 100—indicates the percentage of wheat/acorn flour in the total flour content of the sample; ^a, b, c, …^—mean values within a column marked with different superscript letters indicate statistically significant differences at *p* ≤ 0.05, as determined by Duncan’s test.

**Table 7:** Nutritional value of wheat and wheat–acorn breads, expressed as contents of basic compounds (g/100 g of bread).

Nutrient	Bread Samples	
	W100 + A0	W70 + A30
Carbohydrate	46.46	40.73
Protein	8.69	6.31
Fat	1.25	1.48
Ash	0.34	0.58
Fiber	2.10	4.43

W—wheat flour; A—acorn flour; W100/70 or A0/30—indicates the percentage of wheat flour or acorn flour in the total flour content of the sample.

## Data Availability

The original contributions presented in the study are included in the article. Further inquiries can be directed to the corresponding author.

## References

[1] Oyeyinka S.A., Bassey I.A.V. (2023). Composition, functionality, and baking quality of flour from four brands of wheat flour. J. Culin. Sci. Technol..

[2] Dziki D., Krajewska A., Findura P. (2024). Particle size as an indicator of wheat flour quality: A review. Processes.

[3] Das R.S., Tiwari B.K., Garcia-Vaquero M., Garcia-Vaquero M., Pastor K., Orhun G.E., McElhatton A., Rocha J.M.F. (2023). The Fundamentals of bread making: The science of bread. Traditional European Breads. An Illustrative Compendium of Ancestral Knowledge and Cultural Heritage.

[4] Sun X., Koksel F., Scanlon M.G., Nickerson M.T. (2022). Effects of water, salt, and mixing on the rheological properties of bread dough at large and small deformations: A review. Cereal Chem..

[5] Baráth N., Ungai D.K., Kovács B. (2023). Overview of test methods used to classify wheat flour and bread samples–REVIEW. Acta Agrar. Debreceniensis.

[6] Ajo R.Y. (2018). Effect of acorn flour substitution on Arabic bread properties. Pak. J. Agric. Sci..

[7] Torabi S., Mohtarami F., Dabbagh Mazhary M.R. (2020). The influence of acorn flour on physico-chemical and sensory properties of gluten free biscuits. Food Sci. Technol..

[8] Beltrão Martins R., Nunes M.C., Ferreira L.M.M., Peres J.A., Barros A.I.R.N.A., Raymundo A. (2020). Impact of acorn flour on gluten-free dough rheology properties. Foods.

[9] Silva S., Costa E.M., Borges A., Carvalho A.P., Monteiro M.J., Pintado M.M.E. (2016). Nutritional characterization of acorn flour (a traditional component of the Mediterranean gastronomical folklore). J. Food Meas. Charact..

[10] Akcan T., Gökçe R., Asensio M., Estévez M., Morcuende D. (2017). Acorn (*Quercus* spp.) as a novel source of oleic acid and tocopherols for livestock and humans: Discrimination of selected species from Mediterranean forest. J. Food Sci. Technol..

[11] Al-Rousan W.M., Ajo T.Y., Al-Ismail K.M., Shaker R.R., Osaili T.M. (2013). Characterization of acorn fruit oils extracted from selected Mediterranean *Quercus* Species. Grasas Aceites.

[12] Hrušková M., Švec I., Kadlčíková I. (2019). Effect of chestnut and acorn flour on wheat/wheat-barley flour properties and bread quality. Int. J. Food Stud..

[13] Korus J., Witczak M., Ziobro R., Juszczak L. (2015). The influence of acorn flour on rheological properties of gluten-free dough and physical characteristics of the bread. Eur. Food Res. Technol..

[14] Purabdolah H., Sadeghi A., Ebrahimi M., Kasheninejad M., Tabarestani H.S., Mohamadzadeh J. (2020). Techno-functional properties of the selected antifungal predominant LAB isolated from fermented acorn (*Quercus persica*). J. Food Meas. Charact..

[15] Švec I., Hrušková M., Kadlčíková I. (2018). Features of flour composites based on the wheat or wheat-barley flour combined with acorn and chestnut. Croat. J. Food Sci. Technol..

[16] Gonzaga M., Batista M., Correia P., Guiné R. Development and characterization of wheat breads with acorn flour. Proceedings of the ICEUBI2015—International Conference of Engineering: Engineering for Society.

[17] Park J.Y., Joo J.I., Kim J.M. (2017). Changes in the quality changes of bread added with acorn flour during the storage periods. J. East Asian Soc. Diet. Life.

[18] Skendi A., Mouselemidou P., Papageorgiou M., Papastergiadis E. (2018). Effect of acorn meal-water combinations on technological properties and fine structure of gluten-free bread. Food Chem..

[19] Mousavi B., Ghaderi S., Hesarinejad M.A., Pourmahmoudi A. (2021). Effect of varying levels of acorn flour on antioxidant, staling and sensory properties of Iranian toast. Int. J. Food Stud..

[20] Rashid R.M.S., Sabir D.A., Hawramee O.K. (2014). Effect of sweet acorn flour of common oak (*Quercus aegilops* L.) on locally Iraqi pastry (kulicha) products. J. Zankoy Sulaimani-Part A.

[21] Korus A., Gumul D., Krystyjan M., Juszczak L., Korus J. (2017). Evaluation of the quality, nutritional value and antioxidant activity of gluten—Free biscuits made from corn—Acorn flour or corn– hemp flour biscuits. Eur. Food Res. Technol..

[22] Beltrão Martins R., Gouvinhas I., Nunes M.C., Ferreira L.M.M., Peres J.A., Raymundo A., Barros A.I.R.N.A. (2022). Acorn flour from holm oak (*Quercus rotundifolia*): Assessment of nutritional, phenolic, and technological profile. Curr. Res. Food Sci..

[23] AOAC Official Methods (2023). Official Methods of Analysis of Association of Official Analytical Chemists.

[24] ICC Standard Methods (2001). International Association for Cereal Science and Technology (ICC).

[25] (1999). AACC Method 56-20.01. Hydration capacity of pregelatinized cereal products. Approved Methods of Analysis.

[26] Sobczyk A., Pycia K., Jaworska G., Kaszuba J. (2017). Comparison of fermentation strength of the flours obtained from the grain of old varieties and modern breeding lines of spelt (*Triticum aestivum* ssp. spelta). J. Food Process. Preserv..

[27] (2016). Cereal Grains and Cereal Preparations—Determination of Stickiness of Flour—Method Using an Amylograph.

[28] (2015). Wheat Flour—Physical Characteristics of Dough. Part 1. Determination of Water Absorption and Rheological Properties Using a Farinograph.

[29] (2015). Wheat Flour—Physical Characteristics of Dough. Part 1. Determination of Water Absorption and Rheological Properties Using an Extensograph.

[30] Mohammadi M., Sadeghnia N., Azizi M.H., Neyestani T.R., Mortazavian A.M. (2014). Development of gluten-free flat bread using hydrocolloids: Xanthan and CMC. J. Ind. Eng. Chem..

[31] Cappelli A., Oliva N., Cini E. (2020). A systematic review of gluten-free dough and bread: Dough rheology, bread characteristics, and improvement strategies. Appl. Sci..

[32] Correia P.R., Nunes M.C., Beirão-da-Costa M.L. (2013). The effect of starch isolation method on physical and functional properties of Portuguese nut starches. II. Q. rotundifolia Lam. and Q. suber Lam. acorns starches. Food Hydrocoll..

[33] Taib M., Bouyazza L. (2021). Composition, physicochemical properties, and uses of Acorn starch. J. Chem..

[34] Zhao T., Zhang H., Chen F., Tong P., Cao W., Jiang Y. (2022). Study on structural changes of starches with different amylose content during gelatinization process. Starch-Stärke.

[35] Cappai M.G., Alesso G.A., Nieddu G., Sanna M., Pinna W. (2013). Electron microscopy and composition of raw acorn starch in relation to in vivo starch digestibility. Food Funct..

[36] Irinislimane H., Belhaneche-Bensemra N. (2017). Extraction and characterization of starch from oak acorn, sorghum, and potato and adsorption application for removal of Maxilon Red GRL from wastewater. Chem. Eng. Commun..

[37] Donmez D., Pinho L., Patel B., Desam P., Campanella O.H. (2021). Characterization of starch–water interactions and their effects on two key functional properties: Starch gelatinization and retrogradation. Curr. Opin. Food Sci..

[38] Huang Q., Chen X., Wang S., Zhu J., Wang S. (2020). Amylose–Lipid Complex. Starch Structure, Functionality and Application in Foods.

[39] Cacak-Pietrzak G., Sułek A., Wyzińska M. (2019). Evaluation the baking value of passage flours. Res. Rural Dev..

[40] Saleh M., Taibi A., AlKhamaiseh A.M. (2023). Effect of wheat bran levels and particle size on the rheological properties of wheat flour dough. Jordan J. Agric. Sci..

[41] Cacak-Pietrzak G., Dziki D., Gawlik-Dziki U., Parol-Nadłonek N., Kalisz S., Krajewska A., Stępniewska S. (2023). Wheat bread enriched with black chokeberry (*Aronia melanocarpa* L.) pomace: Physicochemical properties and sensory evaluation. Appl. Sci..

[42] Miś A., Krekora M., Niewiadomski Z., Dziki D., Nawrocka A. (2020). Water redistribution between model bread dough components during mixing. J. Cereal Sci..

[43] Verbeke C., Debonne E., Versele S., Van Bockstaele F., Eeckhout M. (2024). Technological evaluation of fiber effects in wheat-based dough and bread. Foods.

[44] Yavuz Z., Törnük F., Durak M.Z. (2021). Effect of oleaster flour addition as a source of dietary fiber on rheological properties of wheat dough. Eur. Food Sci. Eng..

[45] Xu J., Li Y., Zhao Y., Wang D., Wang W. (2021). Influence of antioxidant dietary fiber on dough properties and bread qualities: A review. J. Funct. Foods.

[46] Yazar G., Kokini J.L., Smith B. (2024). Impact of endogenous lipids on mechanical properties of wheat gluten fractions, gliadin and glutenin, under small, medium, and large deformations. Lipidology.

[47] Dapčević Hadnađev T., Pojić M., Hadnađev M., Torbica A., Akyar I. (2011). The role of empirical rheology in flour quality control. Wide Spectra of Quality Control.

[48] Mirza Alizadeh A., Peivasteh Roudsari L., Tajdar Oranj B., Beikzadeh S., Barani Bonab H., Jazaeri S. (2022). Effect of flour particle size on chemical and rheological properties of wheat flour dough. Iran. J. Chem. Chem. Eng..

[49] Ma W., Yu Z., She M., Zhao Y., Islam S. (2019). Wheat gluten protein and its impacts on wheat processing quality. Front. Agric. Sci. Eng..

[50] Abd-El-Khalek M. (2020). Combined effect of vital wheat gluten, ascorbic acid and emulsifier addition on the quality characteristics of whole grain barley bread. SVU Int. J. Agric. Sci..

[51] Forouhar A., Saberian H., Kaykha M.E.H. (2024). Optimization of producing baguette bread containing acorn flour and evaluating its characteristics. Iran. J. Food Sci. Ind..

[52] Hu X., Cheng L., Hong Y., Li Z., Li C., Gu Z. (2021). Combined effects of wheat gluten and carboxymethylcellulose on dough rheological behaviours and gluten network of potato–wheat flour-based bread. Int. J. Food Sci. Technol..

[53] Levent A., Aktaş K. (2024). Nutritional composition and staling properties of gluten-free bread-added fermented acorn flour. Food Sci. Nutr..

[54] Torbica A., Belović M., Popović L., Čakarević J., Jovičić M., Pavličević J. (2021). Comparative study of nutritional and technological quality aspects of minor cereals. J. Food Sci. Technol..

[55] Sujka K., Koczoń P., Ceglińska A., Reder M., Ciemniewska-Żytkiewicz H. (2017). The application of FT-IR spectroscopy for quality control of flours obtained from Polish producers. J. Anal. Methods Chem..

[56] Mao X., Chen W., Huyan Z., Sherazi S.T.H., Yu X. (2020). Impact of linolenic acid on oxidative stability of rapeseed oils. J. Food Sci. Technol..

[57] Lu Y., Zhao J., Xin Q., Yuan R., Miao Y., Yang M., Mo H., Chen K., Cong W. (2024). Protective effects of oleic acid and polyphenols in extra virgin olive oil on cardiovascular diseases. Food Sci. Hum. Wellness.

[58] Ajo R., Al-Rousan W.M., Rababah T.M., Maghaydah S., Angor M.M., Alomari D., Bartkute-Norkuniene V. (2020). Physiochemical properties and nutritional profile of Mediterranean oak acorn. Afr. J. Food Agric. Nutr. Dev..

[59] Rybicka I., Gliszczyńska-Świgło A. (2017). Minerals in grain gluten—Free products. The content of calcium, potassium, magnesium, sodium, copper, iron, manganese and zinc. J. Food Compos. Anal..

